# 2271. Suppressing the Penicillin-Cephalosporin Allergy Cross-Reactivity Alert: Safety and Impact on Cephalosporin Prescribing

**DOI:** 10.1093/ofid/ofad500.1893

**Published:** 2023-11-27

**Authors:** Lauren Russell, Tamara Krekel, Elizabeth Neuner, David J Ritchie, Helen Newland, Emily J Owen, Alice Bewley, Michael J Durkin, Kevin Hsueh, Jennifer Monroy, Brian C Bohn

**Affiliations:** Barnes-Jewish Hospital, St. Louis, Missouri; Barnes-Jewish Hospital, St. Louis, Missouri; Barnes-Jewish Hospital, St. Louis, Missouri; Barnes-Jewish Hospital, St. Louis, Missouri; BJC HealthCare, St. Louis, Missouri; Barnew-Jewish Hospital, Saint Louis, Missouri; Washington University School of Medicine in St. Louis, St. Louis, Missouri; Washington University School of Medicine, St. Louis, Missouri; Washington University in St. Louis, St. Louis, MO; Washington University School of Medicine, St. Louis, Missouri; T2 Biosystems, Inc., Eureka, Missouri

## Abstract

**Background:**

Beta-lactam antibiotics are the preferred therapy for many infections. Alternative or broad-spectrum antibiotics are often prescribed to patients with documented penicillin allergies, which can lead to increased rates of mortality, hospital-acquired infections, antimicrobial resistance, surgical site infections, hospital length of stay, and cost of care. In March 2021, BJC health-system modified and suppressed the penicillin-cephalosporin allergy cross-reactivity alert for non-serious allergic reactions and implemented an alert prompting clarification of documented penicillin allergies without specific reactions documented.

**Methods:**

This quasi-experimental, retrospective study includes patients with a documented penicillin allergy and a systemic antibiotic order prescribed for outpatient encounters or administered for inpatient encounters from March 2019-February 2020 (pre-intervention) or March 2021-February 2022 (post-intervention) at Barnes-Jewish Hospital or St. Louis Children’s Hospital. Patients with a baseline documented cephalosporin or carbapenem allergy were excluded. Descriptive statistics, chi-squared, or Mann-Whitney U tests were utilized as appropriate. The primary outcome was cephalosporin use and the main secondary outcome was allergic reaction to the cephalosporin ordered or administered.

**Results:**

The pre and post-intervention groups consisted of 7,765 and 7,361 encounters with 25,141 and 24,142 systemic antibiotic orders, respectively. Cephalosporin prescribing was increased post alert suppression by 5.5% (27.7% pre-intervention vs. 33.2% post-intervention, p< 0.001) (Table 1). Allergic reactions after cephalosporin use were not increased post-intervention (Table 2). Out of 1,324 alerts to improve allergy documentation in patients with an “other” or “unknown” penicillin allergy, 4.2% resulted in penicillin allergy documentation updates (Table 3).

**Table 1.**
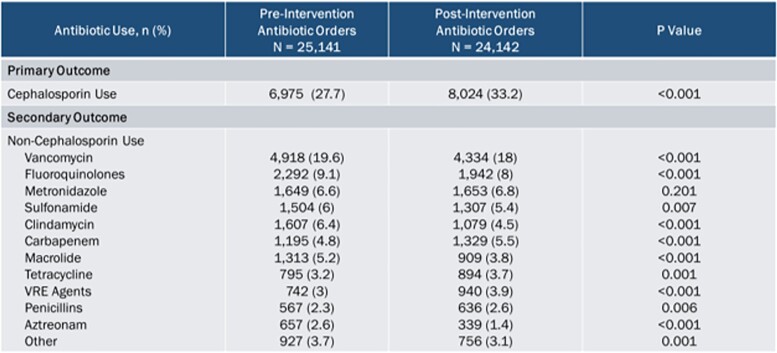
Antibiotic Use

Cephalosporin use was increased post alert suppression by 5.5%, p<0.001. Use of many alternative antibiotics, including vancomycin, fluoroquinolones, macrolides, clindamycin, aztreonam decreased in the post-intervention; however, use of carbapenems, tetracyclines and vancomycin resistant Enterococcus agents increased.

**Table 2.**
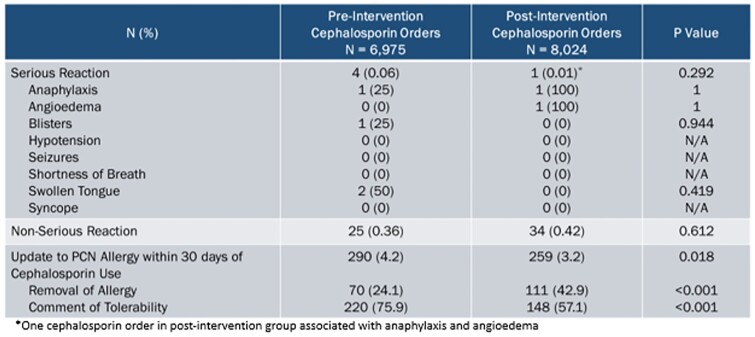
Allergic Reactions to Cephalosporins

Allergic reactions to the ordered or administered cephalosporin, as identified by addition of a new allergy within 30 days to the electronic medical record, was similar between pre-intervention and post-intervention groups. Only 5/14,999 (0.03%) and 59/14,999 (0.4%) cephalosporin orders were associated with serious and non-serious allergies, respectively. Fewer orders in the post-intervention group were associated with updates to penicillin allergy documentation; however, more orders were associated with removal of penicillin allergies in the post-intervention group, p<0.001.

**Table 3.**
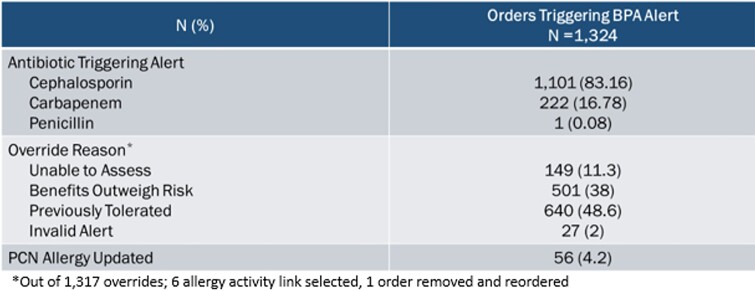
BestPractice Advisory Alert Outcomes

The BestPractice Advisory (BPA) alert appeared for prescribers when ordering a cephalosporin, carbapenem, or penicillin group antibiotic in a patient with a documented “unknown” or “other” penicillin allergy to improve allergy documentation. From this alert, the prescriber can keep or remove the order or select a hyperlink to update the penicillin allergy. If the order was kept, the prescriber had to select an override reason. The penicillin allergy was updated during hospitalization for 55 inpatient alerts and within 30 days for 1 outpatient alert.

**Conclusion:**

The results of this study indicate that the suppression of penicillin-cephalosporin cross allergy alerts is safe and effective in increasing cephalosporin prescribing without increased allergic reactions. Other institutions may consider an alert to improve unclear allergy documentation.

**Disclosures:**

**Tamara Krekel, PharmD, BCPS, BCIDP**, AbbVie: Honoraria|Merck: Honoraria|Shionogi: Honoraria **David J. Ritchie, PharmD, BCPS (AQ-ID)**, Abbvie: Honoraria|Shionogi: Honoraria **Emily J. Owen, PharmD, MS, BCPS, BCCCP**, Mallinckrodt: Advisor/Consultant|Mallinckrodt: Speaker's Bureau **Brian C. Bohn, PharmD, BCIDP**, T2 Biosystems, Inc.: Currently employed as a Medical Science Liaison by T2 Biosystems, Inc.

